# Predictive factors of oral mucositis in patients with locally recurrent inoperable head and neck cancer treated with boron neutron capture therapy

**DOI:** 10.2340/1651-226X.2025.44039

**Published:** 2025-09-23

**Authors:** Tanja Mälkiä, Hanna Koivunoro, Tiina Seppälä, Leena Kankaanranta, Liisa Porra, Anu Anttonen, Mikko Tenhunen, Heikki Joensuu

**Affiliations:** a Department of Radiation Oncology, Helsinki University Hospital Comprehensive Cancer Center and University of Helsinki, Helsinki, Finland; b Neutron Therapeutics Finland Oy, Helsinki, Finland

**Keywords:** Boron neutron capture therapy, oral mucositis, head and neck cancer, radiation therapy

## Abstract

**Background and purpose:**

The prognosis of recurrent head and neck (HN) cancer is poor. High response rates have been achieved with boron neutron capture therapy (BNCT) in the treatment of locally recurrent HN cancer. Radiation-induced oral mucositis (OM) is a common adverse effect of BNCT. We evaluated the factors associated with severe OM in patients treated with BNCT.

**Patient/material and methods:**

Ninety two patients with locally recurrent inoperable HN cancer were treated with nuclear reactor-based BNCT in 1–2 fractions. The association between grade 3 OM, patient clinical parameters and maximum weighted oral mucosa dose was evaluated. Mucosal dose was calculated using the skin nitrogen concentration of 4.2%. A sigmoidal normal tissue complication probability (NTCP) model was created by dividing patients into four equal sized groups based on increasing dose levels.

**Results:**

Grade 3 OM was observed in 42% of patients after the first BNCT treatment. The prevalence of OM increased with higher maximum oral mucosa doses. From the NTCP curve, we estimated the dose corresponding to a 50% probability (D_50_) for grade 3 OM to be 13.5 Gy(W). Older age was the only patient-related factor significantly associated with increased grade 3 OM risk.

**Interpretation:**

Higher maximum oral mucosa doses increased the risk of grade 3 OM. Older age was the only other factor related to severe OM.

## Introduction

The treatment of locally recurrent head and neck (HN) cancer remains challenging despite multimodal approaches. Approximately 2/3 of patients present with stage III or IV disease, and the locoregional recurrence rate in advanced cases varies 15–50%, typically occurring within 2 years of primary treatment [1, 2]. Patients with non-metastatic recurrent tumors are treated with curative intent if possible. Salvage surgery is a curative option for a few patients with resectable recurrence, achieving disease control in 15–30% of cases [3–5]. However, for most patients, surgery is not viable, and radiotherapy with or without chemotherapy is used with curative intent. Overall prognosis after recurrence remains poor [6, 7]. Therefore, new treatment modalities are needed.

Boron neutron capture therapy (BNCT) has shown high response rates (58–81%) in clinical studies of locally recurrent HN cancer [8–13]. BNCT is a cell-selective particle radiotherapy based on neutron capture reaction. When a non-radioactive isotope of boron (^10^B) is irradiated with thermal neutrons, it produces a high linear energy transfer (LET) α-particle (^4^He^2+^) and recoils lithium (^7^Li) nuclei [14]. Due to their short range (<10 µm) in tissue, the effect is primarily local and limited to boron-containing cells while preserving normal cells. Tumor-selective boron uptake is achieved using boron carriers such as L-boronphenylalanine (L-BPA). Oral mucosa is considered a highly sensitive organ-at-risk in BPA-mediated BNCT, due to rapidly dividing epithelial cells, and high boron uptake [15].

In a phase I/II trial at the Finnish FiR 1 research reactor, 76% of patients responded to BNCT and 54% developed grade 3 oral mucositis (OM) [9, 11, 16]. This study was designed to evaluate the reasons for the high prevalence of grade 3 OM within the HN patient cohort treated with the reactor to improve future treatments using an accelerator-based BNCT.

## Patients/material and methods

### Study design and objectives

This is a retrospective study of HN cancer patients treated at the FiR 1 reactor during years 2003–2012 [9, 11, 16]. The primary objective was to identify factors associated with grade 3 OM.

### Patients

A total of 102 patients with histologically confirmed, locally recurrent, inoperable HN cancer, without distant metastases and no available effective standard treatment, were treated with BNCT with the intent of local control. Of these, 92 patients were included in the study, 10 were excluded due to early death (*n* = 4) or missing data (*n* = 6). A total of 30 patients were treated within phase I–II study, the rest outside the trial. Most patients had received extensive prior treatments (Table 1). The most common primary tumor site was oral cavity (37%). Median gross tumor volume (GTV) at the time of initial BNCT was 92 cm^3^ (range: 5–660 cm^3^).

**Table 1:** Patient characteristics.

Characteristic	*n* (%)	Median (Range)
**Total patients**	92 (100)	
**Sex**		
Female	42 (46)	
Male	50 (54)	
**Age (years)**		62 (17–81)
**Prior surgery**	69 (75)	
**Prior RT**	88 (96)	
**Number of RT**		
0	4 (4)	
1	75 (82)	
2	13 (14)	
**Cumulative RT dose (Gy)**		65 (24–129.4)
**Concomitant chemotherapy during RT**	33 (36)	
**Chemotherapy before BNCT**	21 (23)	
**Period between RT and BNCT (y)**		1.4 (0.1–23.5)
**Site**		
Oral cavity	34 (37)	
Pharynx (naso, oro)	21 (23)	
Maxillary sinus/paranasal cavity	12 (13)	
Larynx	8 (9)	
Neck	6 (7)	
Parotid gland	4 (4)	
Skull base	4 (4)	
Other (lacrimal gland, esophagus, thyroid)	3 (3)	
**Histology**		
Squamous cell carcinoma	74 (80)	
Adenoid cystic carcinoma	8 (9)	
Sarcoma	6 (7)	
Adenocarcinoma	2 (2)	
Ductal carcinoma	1 (1)	
Neuroblastoma	1 (1)	
**WHO status**		
0	3 (3)	
1	34 (37)	
2	26 (28)	
3	12 (13)	
4	2 (2)	
unknown	15 (16)	

RT: radiotherapy; BNCT: boron neutron capture therapy.

### Treatment planning

For contouring, planning computed tomography (CT) was co-registered with contrast-enhanced T1-weighted magnetic-resonance images (MRI) and optional ^18^F-BPA-positron emission tomography (PET). CT was used to construct the 3D-model. Treatment planning was performed using the Monte Carlo software SERA (Simulation Environment for Radiotherapy Applications; Idaho National Laboratory, Idaho Falls, ID). The SERA system is not compatible for re-segmentation or recalculations anymore, and the FiR 1 beam model is not available in current treatment planning systems (TPS).

The elemental compositions of tissues were defined according to the International Commission on Radiation Units & Measurements (ICRU) Report 46 [17]. The voxel size for dose calculation was 1 cm^3^.

The dose weighted with relative biological effectiveness (D_W_) of BNCT is expressed as the sum of the four dose components: boron, gamma, nitrogen capture and fast neutron dose. Each dose component is multiplied by a constant weighting factor (w_i_), except for the boron dose, which is multiplied with boron carrier and tissue-specific compound biological effectiveness (CBE) factor [18]. D_W_ aims to describe the equivalent photon dose that causes the same effect as BNCT and is expressed in unit Gy(W).


$${\text{D}}_{\text{W}}={\text{w}}_{\text{B}}{\text{D}}_{\text{B}}+{\text{w}}_{\text{g}}{\text{D}}_{\text{g}}+{\text{w}}_{\text{N}}{\text{D}}_{\text{N}}+{\text{w}}_{\text{fast\_n}}{\text{D}}_{\text{fast\_n}}$$ (1)

where D_B_ is boron dose, D_g_ gamma, D_N_ nitrogen capture and D_fast_n_ fast neutron dose.

Weighting factors were 1 for gamma, 3.2 for nitrogen capture and fast neutrons and 3.8 and 2.5 for boron in tumor and mucosa, respectively [19]. Weighted doses to the tumor, target volume and sensitive tissues were calculated based on the average boron concentration in whole blood during irradiation. A boron concentration ratio of 3.5:1 was assumed for tumor-to-blood, and 2:1 for mucosal membrane-to-blood [16].

The dose to the mucosal membrane was one of the dose-limiting factors in BNCT, restricted to 6 Gy or less per treatment, corresponding to approximately 13.5 Gy(W). Tumor dose varied according to safety limits of normal tissues. Mucosal membrane was not contoured in the treatment planning process. Instead, the maximum dose was visually estimated based on the isodose contours calculated on CT images.

Additionally, the maximum weighted dose to the oral mucosa was calculated. As the elemental composition of oral mucosa is not well-established, the dose was estimated using the nitrogen concentration of skin tissue (*N* = 4.2%) [20].

### Treatment

The patients were scheduled to receive two BNCT treatments at 3–5 weeks intervals, and 52% completed both. Irradiation was performed using the 250 kW FiR TRIGA Mark II reactor (General Atomics, San Diego, CA), located in Espoo, Finland. L-BPA was manufactured by Interpharma Praha (Prague, Czech Republic).

Patients received a 2-hour intravenous infusion of L-BPA at 400 mg/kg. Blood samples were taken before the start of L-BPA infusion, at approximately 20-minute intervals during and after the infusion, and after irradiation of each field. Blood boron concentration was analyzed using inductively coupled plasma atomic emission spectrometry (ICP-AES). Average boron concentration during irradiation was based on the estimation of pharmacokinetic models [21].

Irradiation began approximately 1.5 hours after completing the L-BPA infusion. It was delivered via one to three fields, typically two opposing lateral or anterior-lateral circularly collimated fields.

Patients received cetirizine hydrochloride 10 mg before L-BPA infusion. Dexamethasone 10–15 mg was administered daily after completion of the infusion to prevent radiation-associated edema and was tapered over 3–4 weeks.

### Follow up and evaluation of OM

Trial patients had a follow-up visit 1 month after BNCT treatment, then every 12 weeks. Patients treated outside of the trial did not have a specific follow-up schedule. OM data in this study were obtained within 1 month after the first treatment. If the patients underwent two BNCT treatments, only the OM status after the initial treatment was evaluated.

OM is defined as inflammation and ulceration of oral mucosal membranes. OM was assessed using the National Cancer Institute Common Toxicity 3.0 criteria (NCICT 3.0), where grade 3 is defined as confluent ulcerations or pseudomembranes with bleeding with minor trauma.

### Statistical analysis

Association between grade 3 OM and patient characteristics and treatment-related parameters was analyzed using binary logistic regression (IBM SPSS Statistics,Version: 29.0.0.0(241)). The relationship between grade 3 OM and age, and grade 3 OM and the maximum oral mucosa dose, were analyzed using the Kruskal–Wallis test. Statistical significance was set at *p* < 0.05.

A sigmoidal normal tissue complication probability (NTCP) model was created by dividing the patients into four groups (*n* = 23 each) based on the magnitude of the maximum mucosal dose (mean group doses: 9.9, 11.8, 12.8 and 13.9 Gy(W)). The probability of grade 3 OM was calculated for each dose group based on observed responses. As a simple theoretical approach, a sigmoid curve was fitted to the data.


NTCP=11+(D50D)k (2)

where D is the maximum mucosal dose, D_50_ the dose causing 50% probability of grade 3 OM and k the curve steepness.

## Results

**Figure 1:**
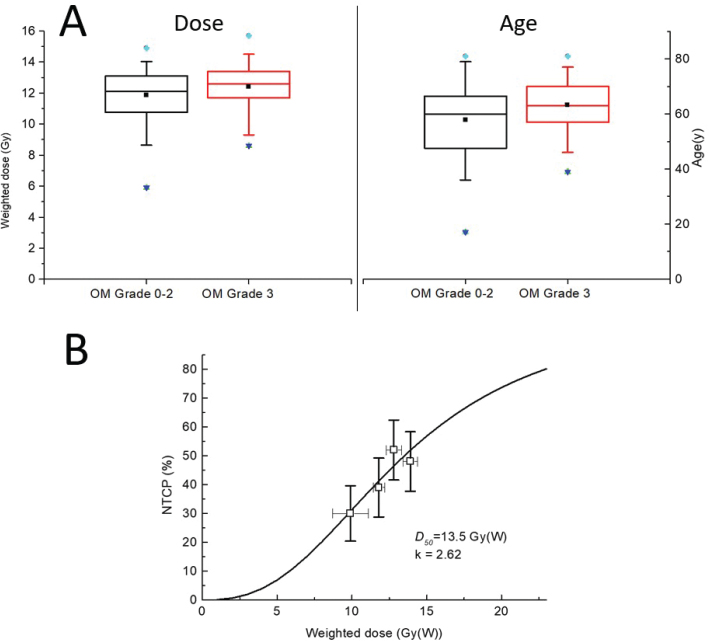
(A) Box plots of maximum oral mucosa dose and grade 3 oral mucositis, and between patient age and grade 3 oral mucositis. (B) Observed normal tissue complication probabilities (NTCP) of Grade 3 OM as mean (open squares) ± SD and the fitted sigmoidal model (solid line) where the 50% complication rate D_50_ = 13.5 Gy(W).

Among 92 patients, 15% had no OM, 15% developed grade 1, 27% grade 2 and 42% grade 3 OM. No grade 4 or 5 events occurred. Among patients (Table 1), only older age was significantly associated with a higher prevalence of grade 3 OM (*p* < 0.032) (Figure 1A). Patients with grade 3 OM had a higher mean age of 63 years (range 39–80 years), compared to 58 years (range 17–81 years) in those with lower-grade OM. No other factors showed a significant correlation with grade 3 OM.

### BNCT oral mucosa dose-response

Maximum oral mucosa doses varied considerably (Table 2). Patients with grade 3 OM had median maximum dose 12.6 Gy(W) (range: 8.6–15.7 Gy(W)). Although the difference in median dose between grade 3 and grade 0–2 OM groups was not statistically significant (*p* = 0.123), a trend toward higher doses was observed in the grade 3 group (Figure 1A). The NTCP curve estimated a D_50_ of 13.5 Gy(W), indicating a 50% probability of grade 3 OM, with a steepness factor *k* = 2.62 (Figure 1B).

**Table 2:** Treatment-related parameters.

Parameter	Grade 0–2 oral mucositis Median (Range)	Grade 3 oral mucositis Median (Range)
Blood boron concentration (µg/g = ppm)	18.8 (11.2–24.9)	18.6 (13.2–26.5)
Time from the end of infusion to the start of 1. field (min)	91 (48–175)	92 (52–146)
Time from the end of infusion to the end of last field (min)	170 (105–257)	171 (118–252)
Time from the beginning of the first field to the end of last field (min)	84 (23–144)	83 (16–157)
Irradiation time (min)	44 (25–72)	43 (22–86)
Maximum oral mucosa dose (Gy(W))	12.1 (5.9–14.9)	12.6 (8.6–15.7)

## Discussion and conclusion

To our knowledge, no previous clinical BNCT studies have reported NTCP-derived D_50_-values for predicting grade 3 OM. An NTCP model by González et al. [22], based on photon radiotherapy patient data from Strigari et al. [23], predicts a somewhat higher D_50_ of approximately 17 Gy.

The median tumor size in present study was 92 cm^3^, with the maximum oral mucosa limited to 13.5 Gy(W). In contrast, Wang et al. and Takeno et al. reported notably lower grade 3 OM rates (29 and 5%, respectively), but their median tumor sizes were substantially smaller – 15.6 and 6.9 cm^3^, respectively [10, 12]. Furthermore, Wang et al. limited the maximum oral mucosa dose to 10 Gy(W) and Takeno et al. to 12 Gy(W), assuming a boron concentration equal to that of blood and a boron dose weighting factor of 4.9. Both continued L-BPA infusion during irradiation, unlike our protocol.

In our study, tumor size and location did not correlate with grade 3 OM. This may be biased by a relatively large irradiated volume (2–3 fields with 11 or 14cm apertures), including nearly entire oral cavity in most cases to reach dose coverage within large GTVs [24].

The prevalence of grade 3 OM was higher among the elderly patients, although the difference was modest. There is no strong evidence in literature regarding the relationship between older age and radiation-induced OM. It is possible that aging can cause slower tissue healing [25, 26].

Severe OM is common in HN radiotherapy (up to 66%) [25]. After all, it is typically reversible and generally considered acceptable.

### Study limitations

This retrospective study has several limitations. Patient heterogeneity and lack of detailed smoking and alcohol history limited risk factor analysis.

The SERA system is no longer available for resegmentation or calculations. The FiR 1 beam model is not available in the current TPS. Due to relatively large irradiation fields, the dose distribution across the mucosal surfaces was fairly homogeneous. Therefore, the primary source of uncertainty might not be the variability in physical dose, but rather the distribution of boron. Average boron concentrations were only available for dose estimation. Although detailed contouring of the mucosal structures was not performed, mucosal dose was evaluated following standard clinical practice.

## Conclusions

Higher maximum oral mucosa doses were associated with an increased risk of grade 3 OM. Older age was the only other risk factor related to severe OM.

## Data Availability

Due to the nature of this research, participants of this study did not agree for their data to be shared publicly, so supporting data are not available.
